# The needs of informal caregivers and barriers of primary care workers toward dementia management in primary care: a qualitative study in Beijing

**DOI:** 10.1186/s12875-018-0890-7

**Published:** 2018-12-20

**Authors:** Meirong Wang, Shuang Shao, Jing Li, Yingjie Liu, Xiaojingyuan Xu, Juan Du

**Affiliations:** 1 School of General Practice and Continuing Education, Capital Medical University, No.10 Xitoutiao, You An Men, Beijing, 100069 China; 2 Dongfeng Community Health Service Center, Chaoyang District, Beijing, China

**Keywords:** Dementia, Primary care, Qualitative approach, Beijing

## Abstract

**Background:**

Informal caregivers of people with dementia in Beijing are increasingly called upon to provide home-based care for their patients due to the increasing number of dementia patients and the shortage of standardized institutional solutions of care for patients in China. This study aimed to clarify the needs of informal caregivers and barriers of primary care workers toward dementia management in primary care in Beijing to provide references that may help to improve the care and services provided to individuals with dementia and their family caregivers residing in urban China.

**Methods:**

A mixed-methods approach was used in this study. We performed individual in-depth interviews with 10 informal caregivers. Moreover, we carried out focus group interviews with 29 primary care workers. Content analysis was used to separately identify themes and codes. Discrepancies were discussed until final agreement was achieved.

**Results:**

Three themes representing the core attitudes of informal caregivers and primary care workers were identified: care knowledge and skills, psychological counseling, and collaborative management. Most primary care workers believed that the management of dementia patients in primary care was necessary. However, due to the heavy work load and different medical specialties involved, these workers were unable to manage it.

**Conclusions:**

Professional training focused on dementia for primary care workers should be strengthened. At the same time, the establishment of a community-based dementia team management model that includes specialists, community health service centers (CHSCs), and community committees should be explored.

## Background

Estimates show that the number of people with dementia will increase from approximately 46.8 million in 2015 to 131 million by 2050 worldwide. In 2016, the global societal economic cost of dementia was estimated to be US$818 billion [1]. This situation challenges governments and health care providers to develop and improve services to people with dementia, with an emphasis on earlier diagnosis, the provision of support in the community, and the role of primary care services [2, 3]. Case management is described as a collaborative process of assessment, planning, facilitation and advocacy for options to meet an individual’s health needs through communication and available resources to promote quality, cost-effective outcomes [4]. Dementia case management interventions are becoming a major way to manage dementia patients in primary care in North America and Europe [5, 6]. A systematic review had demonstrated that some needs (e.g., education about the disease) of patients and caregivers were well targeted by dementia case management, while some very common needs (e.g., early diagnosis) were still overlooked [7]. Furthermore, general practitioners (GPs) often lack knowledge about dementia, and confidence in recognizing the symptoms, they are unsure about how - or if - cognitive screening should be conducted, and often harbor beliefs that little can be done therapeutically in any case [8].

Compared with other countries worldwide, China is experiencing the greatest overall increase in its population of elderly residents. There were approximately 240 million people, or 17.3% of the population, who were aged 60 years or above in 2017 [9]. The number of people in China with dementia is also expected to increase from approximately 9.5 million in 2015 to 16 million by 2030 [1]. There is high-quality evidence suggesting that the total annual cost of dementia will increase from US$900 million in 1990 to US$114.2 billion in 2030 in China [10].

The current medical treatment and management of dementia in China is not promising. Only 10% of people with dementia have been diagnosed, while only 21% diagnosed dementia patients have received medical treatment in urban China [11]. Most people with dementia in China live at home, supported by informal caregivers [12]. The heavy care burden easily leads caregivers to have depression, anxiety and other negative emotions [13], which can cause them to vent their difficult emotions on their patients [14]. But there were also surveys [15, 16] shown that a strong sense of family responsibility might reduce the perception of care burden. Alternative care (respite service, institutional care, etc.) and professional support (professional knowledge and skills guidance, rehabilitation services for dementia patients, psychological support for caregivers, etc.) are important aspects of caregivers’ care needs. However, these needs have not been met effectively [17].

The World Health Organization states that it would be challenging to intervene without the effective involvement of primary care, and dementia should be the priority of public health [18]. However, the national basic package of public health services which was promulgated by National Health and Family Planning Commission of China in 2017 only covered the management of hypertension, diabetes, psychosis and tuberculosis in community health services setting [19]. In 2015, the State Council of the People’s Republic of China promulgated the National Five-year plan for Mental Health (2015–2020), which focused on four brain diseases (autism, schizophrenia, depression and dementia) occurring in different stages of life; the plan includes the development of integrated services, training of mental health specialists, improvement of rehabilitation services and community- and family-based support, as well as the promotion of social awareness [20]. Studies showed that community-care interventions (dietary adjustments, regular home visits, etc.) provided by primary care workers for dementia patients and their caregivers could improve the quality of life for patients and reduce the mental stress of caregivers and the burden of families [21–23]. However, qualified primary care workforces are not sufficient in China. Take GPs for example, there were only 1.82 licensed GPs per 10,000 people in 2017 [24]. Furthermore, a study in Shanghai showed that most GPs still lack training and related knowledge about dementia, and their attitudes toward the care of dementia are pessimistic [25].

As an aging city, there were approximately 150 thousand to 200 thousand dementia patients in Beijing in 2015 [26]. The population of people with dementia is still growing [27]. This qualitative study aimed to clarify the needs of informal caregivers and barriers of primary care workers toward dementia management in primary care in Beijing to provide references that may help to improve the care and services provided to individuals with dementia and their family caregivers residing in urban China.

## Methods

A descriptive approach was chosen because the goal of the study was to elicit the needs of informal caregivers and barriers of primary care workers toward dementia management that would directly and pragmatically show their reality [28].

The topics for the caregivers and primary care workers were designed and modified based on the *expert consensus on long-term healthcare of patients with cognitive disorders in China* by academy of cognitive disorder of China - effective support by caregivers and physicians at lifestyle changes, cognitive maintenance and training [29]. At first the questions were roughly listed based on our literature review and status quo of primary care in China. Then they were categorized by the specific topics that represent the key components of the needs of informal caregivers and barriers of primary care workers’ toward dementia management. We created the topics to ascertain a diversity of perceptions about the needs and barriers mentioned above to make consistent and reliable integration of perspectives from caregivers and primary care workers.

### Participants and recruitment

The study was conducted in four CHSCs in Chaoyang district, Beijing. Twenty-five individuals who were diagnosed with dementia by neurologists in secondary or tertiary hospital were approached by the general practitioner of our research group via electronic health records.

Twenty-five main informal caregivers were approached by the inclusion criteria as follows: (1) minimum 2 h of daily care and the longest duration of care per day for patients at home over 6 months; (2) willingness to be interviewed as part of the study; and (3) could answer the interviewer’s questions clearly and logically.

We purposively sampled primary care workers from the inner city and suburbs with a mix of primary care workers, sexes, ages and years of experience. Inclusion criteria were general practitioner or nurse qualifications, a current primary care practice and at least one patient with dementia.

### Data collection

#### Individual in-depth interviews

In-depth interviews with caregivers were conducted from September to December 2017 by interviewers who received training and supervision in conducting qualitative interviews. The interviews were structured and included predetermined topics and associated probes. These predetermined topics were chosen by the research team to elicit, in an open-ended fashion, an exploration of caregivers’ attitudes. The topics included who else provided support and assistance for you in the community during the care period, what else do you need while providing care, and who would you tend to ask for help if you run into trouble in the process of care. Field notes included difficulties encountered during the interviews and the interviewer’s impressions of participants’ reactions to the study. Participants were advised that the whole interview could be completed in 30 min but were not discouraged from extending the interview duration if willing. The interviews ranged from 30 to 90 min in length, which mostly lasted for approximately one hour.

#### Focus groups

To explore issues identified in the interviews, we conducted focus group with GPs and community nurses from January to February 2018. The objectives were to answer the following questions about primary care workers with respect to dementia: (a) the understanding of knowledge and skills of dementia care; (b) the attitudes, ability and difficulty in dementia management; (c) their advice on dementia care in the community.

After informing participants of the purpose of the study and obtaining written consent, four experienced moderators led the four groups simultaneously. An experienced researcher in qualitative study observed the groups’ discussions and took notes. Each group was given the same task, namely, to identify the barriers to managing dementia in primary care and to suggest effective approaches that could overcome these barriers. Each session was approximately 90 min in length and was held in a private meeting room located in the health clinics.

### Data analysis

All interviews and focus group discussions were audio recorded with consent from participants. Digital recordings were stored in a password-protected, secure system. Audio-taped data were transcribed verbatim. Transcripts were reviewed and analyzed by six members of the research team. To bring a wide variety of knowledge and preconceptions to the analysis process, the team comprised professionals with different research backgrounds, including an associate professor and a lecturer of general practice, three graduate students and a general practitioner. The team members read all the material through several times to obtain the whole sense and then independently coded transcripts to identify themes by condensing and summarizing the contents. When no new topics were identified, data saturation was considered [30].

We conducted a content analysis by analyzing the verbatim transcripts and identifying specific meanings and potential implications, in accordance with the topics. A second researcher conducted a second transcription after a researcher independently transcribed each audiotape. A third researcher then compared the first and second transcriptions for disparities. A final transcription was developed after the researchers collectively discussed the most accurate interpretation of the disparities. No new categories emerged in the analysis after the 8th interview, as interview no. 9 and 10 only added minimal information. Saturation of themes was reached after the fourth focus group and data-collection was stopped. We then reviewed the coded data for different approaches, which led to the combination of some codes, the identification of new subcodes and the production of a narrative summarizing the key themes of the discussions. A back-translation approach was also used, which involved translation to English and then double-checking for errors by a second independent researcher to ensure equivalence in both languages.

## Results

### Sample

According to the severity of the patient’s condition, 12 informal caregivers of people with dementia were selected in Chaoyang district, Beijing. Only 10 caregivers were interviewed; 2 declined because they did not want to talk about their work. Most caregivers were either spouses (6/10, *n* = 6) or adult children (3/10, *n* = 3) (see Table 1 for additional characteristics). The primary care workers were recruited from 8 districts in Beijing, 75% of which were metropolitan. The 29 primary care workers (overall age range, 30–62 years; mean, 38.9 ± 8.3 years) represented a wide range of practice sizes and length of experience (range, 4–29 years; mean, 12.7 ± 6.7 years), and included 20 GPs and 9 community nurses. The ratio of male to female GPs in the groups was 3:17. Twenty GPs were divided into three groups of 7, 8 and 5 GPs; nine community nurses were in a group.

**Table 1:** Characteristics of interviewed caregivers and care recipients

Items	Caregivers (*n* = 10)	Care recipient (*n* = 10)
Mean age	66.9 ± 16.6	79.0 ± 4.1
Sex		
Female	9	2
Male	1	8
Self-reported general health		
Excellent/very good	0	N/A
Good	0	N/A
Fair	3	N/A
Poor	7	N/A
Relationship		
Spouse	6	N/A
Child	3	N/A
Other family relative	1	N/A
Marital status		
Married	9	8
Widowed	1	2
Education		
Elementary school	0	2
Secondary school	6	3
Higher education	4	5
Severity of dementia		
Mild	N/A	5
Moderate	N/A	3
Severe	N/A	2

### Qualitative findings

Analysis of the interviews with the caregivers and the focus groups with primary care workers led to the identification of three themes related to the needs of informal caregivers and barriers of primary care workers: care knowledge and skills, psychological counseling, and collaborative management.

Quotes are shown in italics. “C” stands for caregiver who participated in the interview, “GPA” stands for the first group of general practitioners, “GPB” stands for the second group of general practitioners, “GPC” stands for the third group of general practitioners, and “ND” stands for community nurse.

### Care knowledge and skills

#### Urgent needs for care knowledge

To provide more appropriate care, almost all informal caregivers mentioned that they needed GPs or community nurses to provide professional care guidance. People caring for patients with mild dementia wanted to obtain care guidance about how to prolong the early process of the condition, how to address patients with abnormal behavior problems, how to enhance the patient’s memory and how to improve the patient’s ability to live. People caring for patients with severe dementia wanted to obtain skills to address dietary and toileting difficulties of their patients and wanted to know how to prevent the occurrence of complications or how to address them, etc. They also hoped that the primary care workers could offer home care services, such as injections.

*My father-in-law*’*s condition is not very severe, but we are not good at taking care of him. I hope to grasp some knowledge to prolong the process of the disease.(C8)*

*We need some professional care knowledge because we are not professional. (C7)*

*I learned stomach intubation care from nursing workers when my father was hospitalized. (C6)*

*I take care of my husband. He is bedridden now. I do not know whether it is good to take care of him like before. I am afraid to touch him. (C2)*

*He always gets inflamed. It’s not easy for us to bring him to the CHSC because of his mobility problems. If the medical workers could do some follow-up regularly and offer injection services, it would be more convenient. (C6)*

#### Willing to help but lack professional training

As for the primary care workers, they could provide only a small amount of help because of their heavy workloads and their lack of expertise related to dementia. The help provided to caregivers was mainly focused on the advice regarding usual care for the patients, such as dietary care, the prevention of accidents, and the prevention of complications for bedridden patients. Only three GPs said they had received training related to dementia, but they had already forgotten the specific details. Almost all the interviewees said they only had a preliminary understanding of the knowledge and skills of dementia. For them, the experience mostly came from clinical practice, books, and the web. All interviewees agreed that they lacked confidence regarding the management of dementia patients in primary care.

*I like to help them. I educate the care providers by providing some care knowledge, such as pressure sores care, lung infection care. (N3)*

*I see almost 100 patients every day. It is difficult for me to guide the caregivers particularly. The specialists have a deeper understanding of dementia than us. I think they could provide more targeted advice. (GPB1)*

*As a general practitioner, I pay more attention to the patients’ physical diseases, such as high blood pressure and diabetes, not dementia. (GPC3)*

*The main thing we can do is to provide care for complications during dementia, such as lung infection and high blood pressure. (GPA1)*

*I did not accept any systematic training about dementia during residency. (GPB1)*

*I had managed patients with dementia during residency training of GPs, but my knowledge about dementia is not comprehensive. (GPA1)*

### Psychological counseling

#### Needs for psychological support

The heavy care burden and pressure could easily lead caregivers to irritability, anxiety and other negative emotions. This might lead to a reduction in their ability to provide care and to the quality of the care services provided to the dementia patients. Caregivers need psychological consultations to ease the pressures they felt and to maintain their good emotional state.

*I feel better now. I adjusted myself slowly… I felt helpless and anxious at the beginning. (C10)*

*Sometimes I get angry at my husband. He says he wants to separate from me. He always says something weird like that. (C4)*

*My mum fell in the bathroom a few days ago. Her head wedged in the crack of close stool and pillar. I called her, but she did not respond to me. If I discovered her later, she would never be saved. I am so scared. (C10)*

*His illness really bothers me. My condition was good before, but I feel uncomfortable with my heart recently. He depends on me for everything. I take care of him all day. I am really irritated. (C4)*

*He is stronger than me. He wanted to hit me that day. I was so grieved. (C3)*

*I would be extremely delighted to chat with someone. Nobody talks to me because they are busy. (C1)*

#### Limited help provided by primary care workers

Primary care workers could give informal caregivers only limited psychological guidance and support because of the different medical specialties. Actually, there were already some patients with dementia and their caregivers who had established telephone contact, WeChat (a mobile text and voice messaging communication service) and other contacts with their GPs to accept personalized guidance about psychological problems.

*I think it is still necessary for the specialists to see patients in CHSCs. Sometimes when we say something to the caregivers or patients to express our empathy, they refute us. They think that we cannot understand their feelings. Psychologists have specialized knowledge and they know how to deal with it. (GPB1)*

*Some caregivers are in my WeChat list. If there is any special situation, he can consult me directly on WeChat. I will give him a reply when I have time. I think this is a more personalized guide, but the help I provide is very basic and limited. (GPB2)*

*As a communit*y *health worker, we should increase the public awareness of dementia, help the patients and caregivers as much as possible in various ways. (GPC2)*

### Collaborative management

#### Expectations of respite services

Lack of social contact was a common situation among caregivers. They rarely arranged leisure or social activities, especially those caring for patients who could not take care of themselves. To improve the quality of care, collaboration was necessary. Most informal caregivers were spouses; they were older, and their body functions degenerated gradually. They felt physically overextended and exhausted, and their health condition was becoming gradually worse in the process of taking care of their patients.

*The main thing is that I do not have any time to socialize. I have no energy and I'm tired all the time. (C7).*

*I have to take care of my dad. I really do not have time to take care of my own family. I feel sorry for them. (C6)*

*He likes to go out for a walk, but my body is in a bad situation. I am unable to follow him, and I hope there is someone to follow him. (C1)*

#### Team-based management model

Almost all the primary care workers in the interview indicated that a team-based management model should be established. GPs should be mainly responsible for the early identification and the monitoring of dementia, maintaining the overall physical condition of patients and the overall coordination of the team. Community nurses would undertake care guidance, including life guidance and regular follow-up. In addition, the team also needed to have specialists, such as neurologists or psychiatrists. They could offer professional guidance to the GPs and provide support to the caregivers by seeing patients in CHSCs regularly.

*I think it should be a team. Apart from GPs and community nurses, specialists should be involved according to the patients’ situation and caregivers’ needs. GPs are mainly responsible for the overall physical condition of patients and specialists solve the specific problems. In addition to providing care guidance, community nurses can also do some follow-up. (GPB4)*

As the most accessible social resource for patients, community committees should cooperate with the CHSCs to play an active role in organizing patient exchange activities and other events and in coordinating available social resources to help the patients and their caregivers, such as helping to contact care institutions.

*Community nurses provide some care guidance of patients’ daily life for caregivers, such as prevention of accidental injury. GPs and specialists maintain the patients are in a stable physical condition and provide some psychological support for caregivers. I think this is the best way. It is also good for community committees and care institutions to provide some help because taking care of such patients is not unilateral. More participants, better quality of care work. (N1)*

## Discussion

In this study, two caregivers of people with dementia declined. The possible reasons tended to be either lack of knowledge on the part of the families/patients or the stigma sometimes attached to dementia. There is an overall low level of understanding of the condition of dementia among the public and the nonspecialist domain, which, alongside the stigma that might be attached, makes it difficult for people to discuss. One strategy to enhance attendance and eliminate stigma is by educating society [31]. The public health system in China has been working hard on promoting the social awareness through mental health propaganda and education in hospitals, communities and schools to reduce or eliminate stigma [12]. Since 2012, the appellation of “lao nian xing chi dai” with derogatory meaning in Mandarin has been gradually replaced by “cognitive disorder” in Chinese health care system [32].

People with dementia need help with challenging changes in behavior, memory, physical disability, and mood [33]. Informal caregivers need to know the characteristics of dementia and have knowledge of disease-related therapies and care. The needs for related knowledge persist from the early stages of the patients’ disease; as the disease progresses, the focus of caregivers’ needs change as well. An intervention that examined care skills was carried out in the caregivers of elderly patients with dementia; the research looked at skills that included coping styles, cognitive rehabilitation training and daily life care principles. The results showed that anxiety and depression of the caregivers were relieved and that the life quality score improved after the intervention [34]. Technology such as face-to-face interactions via various secure video connection platforms, and devices that monitor the health status or function of an older adult can be helpful for individuals who are caring for someone at home, especially to those who have heavily care duties and have difficulty leaving the home. In addition, the guidance and application of predictive care can also help to minimize further harm, risk and cost for the patient and the psychological burden on caregivers [35]. In China, GPs are often inadequately trained and may be reluctant or unable to detect, diagnose or manage dementia [36]. The results of the interviews also showed GPs were more focused on the physical diseases of their dementia patients and paid insufficient attention to their dementia, similar to a previous survey conducted in Shanghai, China [25]. Guidelines for the management of dementia in primary care have been established in Australia and Canada [37, 38]. These guidelines are intended to be used by health professionals and policy makers who plan, organize and deliver care for people who have dementia and their caregivers. Therefore, the domestic management of dementia in primary care should focus on strengthening the professional training regarding dementia for medical workers and establishing guidelines that suit the national culture.

Psychological counseling for caregivers is an important part of care. The caregivers’ positive mental attitudes are the basis for the implementation of care. In America, GPs periodically assess caregivers for the level of perceived burden, presence of depression and anxiety, and coping strategies, and they help the patient and caregiver with care planning in advance. Community nurses, social workers, rehabilitation specialists, and psychologists provide support services such as health education, psychological counseling, and emotional support for caregivers [39]. Therefore, given the needs of caregivers in China, it is recommended that this model be used as a reference. Meanwhile, hospitals and CHSCs provide psychological counseling and care guidance for the caregivers collectively and organize gatherings for caregivers on a regular basis to promote the sharing of feelings and care skills. In addition, respite service is necessary according to the caregiver’s work and physical condition to deliver an individualized, simple, flexible, and adaptable response to caregivers’ needs [40].

Community management of dementia involves a wide range of areas, requiring multidisciplinary collaboration. Various social resources in addition to primary care support should also be noted. Community committees, neurologists and psychologists in secondary and tertiary hospitals should be available to collaborate. A clear and robust multidisciplinary expert group will enable diagnosis and management [41] and enhance patient and caregiver satisfaction [42]. This integration of medical and social services, emphasizing multidisciplinary and multidomain team communication and collaboration of the personalized management model, fully embodies the principle of being “patient centered”.

In North America and Europe, case management has become an important way for primary care providers to manage dementia in communities. Case managers are mostly nurses who care for the dementia patients; they work with GPs, assisting physicians in conducting examinations around the diagnosis, coordinating treatment plans, providing health education on diseases, suggesting available resources and reporting the health status and needs of patients and caregivers regularly. The GPs are responsible for providing medical treatment, developing a care plan, and adjusting the plan according to the feedback of the case managers [7]. Domestic case management was first introduced in Taiwan and Hong Kong and has a short development history in the mainland. The current application in community schizophrenia is relatively mature and has achieved a significant effect [43]. Prior work showed that patients’ cognitive ability, self-care ability and quality of life were all improved through the application of dementia case management [44]. Case management not only had a positive impact on dementia patients and caregivers themselves but also improved the utilization of community health resources and reduced the cost of health care [45]. To some extent, services provided will be dependent on local provisions, demography, and geography, but even if the services are available, if the understanding or relationship of expectations between the GPs and specialists is dysfunctional, then patients will not receive the high quality of care that they deserve [46]. At present, the case management of dementia in China is still at an exploratory stage. There is no clear definition of team formation rules, implementation processes of case management and evaluation of management effectiveness. Based on these concerns, the establishment of a community-based dementia team management model that suits the national culture should be explored.

It became clear that there were many similar aspects about the needs of informal caregivers and barriers of primary care workers toward dementia management in primary care between China and some Western countries. More-general expectations were described by informal caregivers (e.g., sharing the responsibility of caring for dementia patients and making joint decisions regarding the care) [47] and primary care workers (e.g., time constraints and lack knowledge about dementia) [48]. Different aspects were also existed. General practitioners in Western countries perceived lack of knowledge of early diagnostic, and some of them seemed to attach stigma to the condition of dementia by assuming that the caregiver/patient did not want a diagnosis until the symptoms were so severe that it was inevitable [48]. However, these contents were not reflected in our study. We think there are two potential reasons. First, the GPs working in CHSCs are generally less educated in China. Only 44.8% GPs had a bachelor degree or above compared with 69.9% physicians in the secondary and tertiary hospitals [49]. The perceived low quality of primary care is a major reason why people prefer to use hospital care. Second, the management of specific chronic diseases required by the government is an important job for CHSCs. Therefore, primary care workers rarely show more initiative on diseases not required by the government.

After the researchers collectively discussed the most accurate interpretation of the disparities, a final transcription was developed to make consistent and reliable integration of perspectives from caregivers and primary care workers, which increases the confidence in our findings. But this study has its limitations. It is an exploratory study, only the informal caregivers in Chaoyang district were recruited. The representativeness of informal caregivers in this study was relatively limited. We plan to do further studies in other districts of Beijing.

## Abbreviations

C: Caregiver who participated in the interview
CHSCs: Community Health Service Centers
GPA: The first group of general practitioners
GPB: The second group of general practitioners
GPC: The third group of general practitioners
GPs: General practitioners
N: Community nurse
